# 2567 OHRP meets ToS: Cloud-based technologies in human subject research

**DOI:** 10.1017/cts.2018.294

**Published:** 2018-11-21

**Authors:** Assya Pascalev

**Affiliations:** Georgetown - Howard Universities

## Abstract

OBJECTIVES/SPECIFIC AIMS: To identify new ethical challenges in human subject research related to the use of cloud-based platforms for data collection. METHODS/STUDY POPULATION: Ethical analysis. RESULTS/ANTICIPATED RESULTS: The OHRP regulations protecting the data, privacy and confidentiality of human subjects and the Terms of Service regulations governing data use by cloud-based platforms are vastly different. The gap between these 2 sets of laws and regulations leaves human subjects vulnerable to harm during the data collection process via clod-based tools. DISCUSSION/SIGNIFICANCE OF IMPACT: Recognizing the risks related to data gathering via cloud-based platforms, and educating researchers and research subjects about these risks and how to minimize them will strengthen the protections of participants and will enhance the informed consent process resulting in increased trust and greater willingness to participate in human subject research.

